# Association between changed self-rated health and the risk of venous thromboembolism in Malmö Preventive Program: a cohort study

**DOI:** 10.1007/s11239-023-02933-4

**Published:** 2024-01-24

**Authors:** Peter Nymberg, Veronica Milos Nymberg, Susanna Calling, Gunnar Engström, Peter Svensson, Johan Elf, Bengt Zöller

**Affiliations:** 1 School of Health and Welfare, Halmstad University, Halmstad, Sweden; 2 Center for Primary Health Care Research, Department of Clinical Sciences Malmö, Lund University, Region Skåne, Sweden; 3 Center for Thrombosis and Haemostasis, Department of Haematology, Skåne University Hospital, Malmö, Sweden

**Keywords:** Self-rated health, Venous thromboembolism, Cohort studie

## Abstract

Poor self-rated health (SRH) is associated with incident arterial cardiovascular disease in both sexes. Studies on the association between SRH and incident venous thromboembolism (VTE) show divergent results in women and no association in men. This study focuses on the association between change in SRH and incident VTE in a cohort of 11,558 men and 6682 women who underwent a baseline examination and assessment of SRH between 1974 and 1992 and a re-examination in 2002–2006. To investigate if changes in SRH over time affect the risk of incident VTE in men and women. During a follow-up time from the re-examination of more than 16 years, there was a lower risk for incident VTE among women if SRH changed from poor at baseline to very good/excellent (HR 0.46, 95% CI 0.28; 0.74) at the re-examination. Stable good SRH (good to very good/excellent at the re-examination, HR 0.60, 95% CI 0.42; 0.89), or change from good SRH at baseline into poor/fair at the re-examination (HR 0.68, 95% CI 0.51; 0.90) were all significantly associated with a reduced risk for VTE. All comparisons were done with the group with stable poor SRH. This pattern was not found among men. Regardless of a decreased or increased SRH during life, having an SRH of very good/excellent at any time point seems to be associated with a decreased risk of VTE among women.

**Supplementary Information:**

The online version contains supplementary material available at 10.1007/s11239-023-02933-4.

## Highlights

- In this cohort, men had a higher incidence of VTE compared with women.
- A good SRH anytime during life is associated with a lower risk of VTE in women.
- There was no association between the risk of VTE and SRH nor between VTE and changed SRH among men.
- The role of changed SRH regarding different incident diseases should be further investigated.

## Background

Venous thromboembolism has several major risk factors, and some of these have been found to be associated with poor self-rated health (SRH), such as high body mass index (BMI) [1], smoking [2] and physical inactivity [3].

Poor SRH is associated with premature mortality and numerous morbidities [4, 5], which can only partly be explained by the patient’s medical history, cardiovascular risk factors, and socioeconomic characteristics [6, 7]. Studies have also described an association between poor SRH and altered biological markers such as heart rate, glycemic status, and inflammation [8].

Several longitudinal studies of individual changes in SRH over time have shown that SRH seems to be a stable indicator of incident disease, especially in elderly individuals [9, 10], even though the subjective evaluation of SRH among elderly patients may not reflect the decline in objectively measured health. This might be explained by an adaptation to worsening physical conditions with increasing age [11].

However, the relationship between poor SRH and VTE has not been well studied. Previous studies have shown contradictory results with a significant association between poor SRH and VTE only among women [12, 13]. Meanwhile, even if sex differences regarding VTE incidence remain controversial, it has been suggested that biological mechanisms might contribute to the increased incidence for men [14].

The aim of the present study was to investigate whether changes in SRH over the life-course are related to incident VTE in Malmö Preventive Programme of 33 346 individuals.

## Methods

### Study population

Men and women aged 35–70 in the urban area of Malmö, southern Sweden, were invited to participate in a screening programme initiated by the Section of Preventive Medicine, Department of Medicine, University Hospital with a start in 1974, Malmö Preventive Project (MPP). A total of 22,444 men and 10,902 women were included between 1974 and 1992, with an overall attendance rate of 71% [15–17].

A total of 11,558 men and 6682 women attended the re-examination between 2002 and 2006 [18]. The median time between baseline and re-examination was 25.5 years for men and 19.6 years for women. All participants were traced in validated national registers [19] until December 31st, 2018. The median follow-up time from the re-examination was 12.8 years for men and 13.6 years for women.

### Measurements and definitions

SRH at baseline between 1974 and 1992 was assessed by a dichotomous question: ‘Do you feel perfectly healthy? Yes/No’. The answers were translated into good/poor SRH. At the re-examination between 2002 and 2006, a single question with five answering alternatives was used; very poor, poor, good, very good, and excellent. In the present study, based on the distribution, we dichotomized the SRH at re-examination into poor/fair (very poor, poor, and good) and very good/excellent (very good and excellent). In addition to the question about their perceived health, the re-examination questionnaire included data on reported alcohol consumption, smoking, leisure time physical activity, medication, diseases, measures of hip circumference, waist circumference, and experienced symptoms. Data about VTE diagnoses (Supplementary Table 1), and other diagnoses for exclusions were collected using ICD diagnostic codes from the Swedish Hospital Discharge Register and the Hospital Outpatient Register, and data about cancer diagnoses were retrieved from the Swedish Cancer Register [20–22].

### Confounders

We adjusted the multivariate analysis with the following confounders: age [23], smoking [14, 24, 25], varicose veins [26] and waist circumference [27, 28], as waist circumference is a better predictor for VTE than BMI [29, 30].

### Statistical analysis

The stratification between the sexes was confirmed by an interaction analysis. Baseline characteristics and VTE incidence rates for the studied population were calculated using descriptive statistics. The associations between SRH change and VTE were calculated by Cox proportional regression analyses with hazard ratios (HR) and 95% confidence intervals (CI). The analyses were done in several steps, starting with a univariate analysis to examine the hazard for each different potential confounder. In the multivariate analysis, we added the potential confounders that were significantly associated with VTE in three steps. STATA 16.1 was used to perform all analyses.

### Exclusion

A total of 3578 men and 1604 women were excluded from the main analysis due to prevalent disease associated with increased risk for VTE before re-examination [31–33].

## Results

There were 7937 men with 354 incident VTE cases and 5066 women with 269 incident VTE cases during a follow-up time from the re-examination of 16.2 years (men) and 16.8 years (women). The sum of the follow-up time for men was 76 745.602 person-years and 55 842.891 person-years for women, corresponding to an incidence rate of 4.61 per 1000 person-years (95% CI 4.51; 5.12) for men and 4.82 per 1000 person-years (95% CI 4.27; 5.42) for women.

**Fig. 1:**
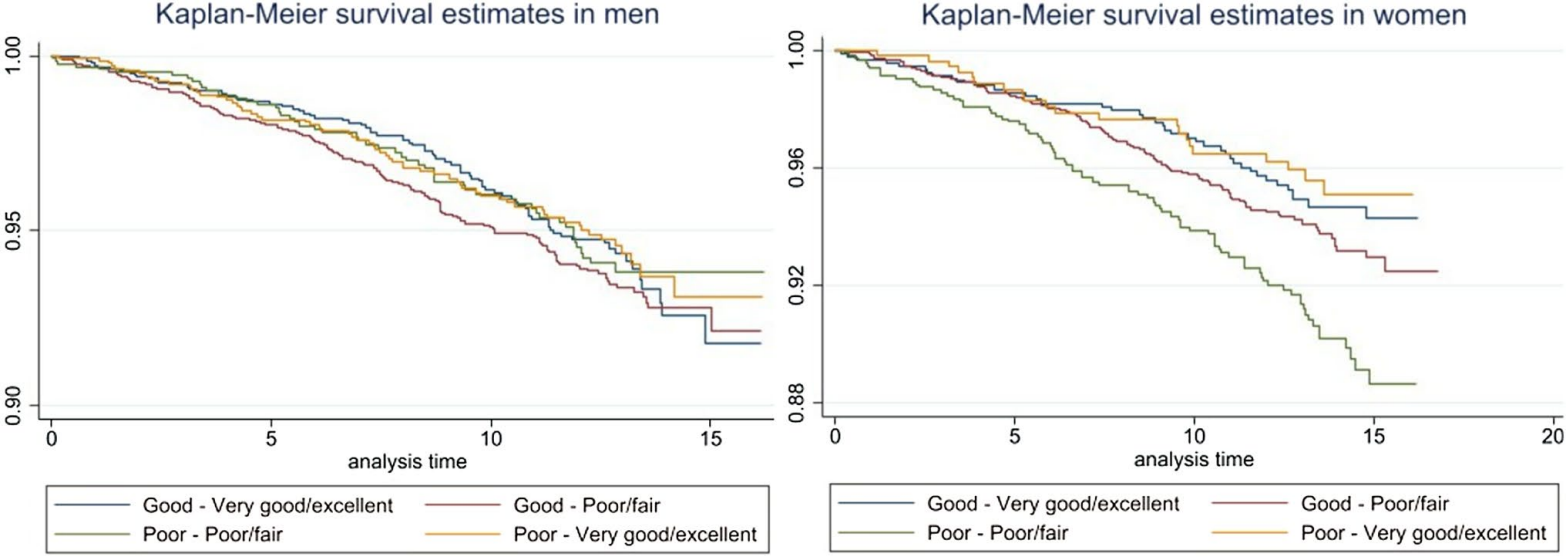
Unadjusted Kaplan-Meier survival estimates for women and men during follow-up. The different lines represent the change in SRH between baseline and re-examination in men (left) respectively in women (right)

The interaction test showed a significant difference between sex and SRH with a p-value < 0.05. A group comparison analysis showed significant differences between the groups with incident and non-incident VTE among women only at baseline regarding SRH (p = 0.007) (supplementary Table 2). Stable good SRH, or good SRH at either baseline or re-examination were all associated with a significantly reduced risk for VTE, compared with rating the health as poor at both baseline and re-examination (Fig. 1).

After an adjustment for age, all SRH change groups were associated with a significantly reduced risk for VTE (Table 1). The association remained significant even when the analysis was fully adjusted with age, smoking, waist circumference, and varicose veins (Table 1).

**Table 1:** Multivariate Cox regression with hazard ratios and adjustments for confounding variables

Model	1	2	3
SRH change			
Men			
Good to Very good/excellent	1.04 (95% CI .74; 1.47)	1.09 (95% CI .77; 1.55)	1.16 (95% CI .81; 1.65)
Good to Poor/fair	1.14 (95% CI .84; 1.56)	1.17 (95% CI .85; 1.61)	1.20 (95% CI .88; 1.68)
Poor to Very good/excellent	1.01 (95% CI .70; 1.46)	1.03 (95% CI .71; 1.49)	1.06 (95% CI .75; 1.59)
Poor to Poor/fair	Ref	Ref	Ref
Total no	7937	7742	7711
Failures no	354	343	341
SRH change			
Women			
Good to Very good/excellent	.55 (95% CI .38; .80)	.55 (95% CI .37; .80)	.60 (95% CI .42; .89)
Good to Poor/fair	.65 (95% CI .49; .85)	.67 (95% CI .50; .87)	.68 (95% CI .51; .90)
Poor to Very good/excellent	.48 (95% CI .30; .79)	.52 (95% CI .27; .75)	.52 (95% CI .32; .86)
Poor to Poor/fair	Ref	Ref	Ref
Total no	4990	4887	4869
Failures no	265	259	259
Adjustments	Age	Age, smoking	Age, smoking, waist circumference, varicose veins
Proportional hazard assumption, global test Men	.4013	.2452	.5098
Proportional hazard assumption, global test Women	.9978	.9647	.9876

Exclusions of patients at baseline with prevalent aortic aneurysm, aortic dissection, atrial fibrillation or flutter event, all peripheral artery disease, arterial thrombosis and embolism, deep venous thrombosis, pulmonary embolism, portal vein thrombosis, superficial thrombophlebitis, malignancy, aortic stenosis, coronary event, stroke, heart failure and individuals treated with warfarin medication

In the sensitivity analysis to test the robustness of the analysis, the association between SRH change and VTE remained significant, using prevalent VTE, prevalent malignancy, and medication with Warfarin as exclusion criteria (Supplementary Table 4 & 5).

Regardless of exclusions, there was no association between change in SRH and incident VTE among men, neither in the univariate analysis nor after the fully adjusted analysis.

## Discussion

The lowest risk for incident VTE was found in women whose SRH changed from poor to very good/excellent. However, we found an association with a 32% lowered risk for VTE among those women who had changed their SRH from good to poor/very poor. This means that women who once rated their health as good had a lower risk for VTE compared with those women who continuously rated their health as poor throughout their lives.

The results differ from studies with CVD, where rating SRH as poor is associated with an increased risk of CVD [5, 34] as well as with mortality [35, 36] in both men and women. Even if the prevalence of VTE is higher among men [37], our results might be explained by the fact that women generally report poorer health than men [38]. This is in line with previous studies showing that despite the subjective assessment of health is poorer among women, they are healthier [39, 40] and have longer life expectancy than men [40, 41].

The presence of economic crises in society may be one explanation for the changes in SRH, where more women than men seem to be affected negatively [42]. Recent research has also claimed that personal finances can disrupt the assessment of one’s self-rated health [43] and should be adjusted for. The re-examination was made between 2002 and 2006, when the unemployment rate was lower compared to during the economic crisis in Sweden during 1990–1994 [44] and, prior to the economic crisis causing a recession period in Europe between 2008 and 2011 [42]. Therefore, the self-reported SRH at the re-examination should not be affected by major societal changes.

## Strengths and limitations

The lack of socioeconomic data or hospitalizations is a limitation of the study. Another limitation is the lack of psychological status assessment and personality traits, which can also influence the experienced SRH [45, 46].

A further limitation of the study is the variation in SRH assessment in baseline and re-examination. However, as SRH is considered a stable indicator over time, we believe that this had a minor impact on our results.

A study on long-term trajectories of SRH showed that a decline in SRH had already started before the diagnosis leading to subsequent death [47]. Our study showed that rating SRH as good at least once during the follow-up time lowers the risk for incident VTE and is, therefore, a potentially useful indicator for early efforts to assess a patient’s risk for consequent disease among women. As around 23% of women in Sweden rate their health as not good [48], it may have a considerable impact on the incidence of VTE. Besides the association between SRH and VTE among women found in our study, SRH has also been found to be associated with altered biological markers [8] and of importance for the development of incident VTE [49].

## Conclusion

Regardless of a decreased or increased SRH during life, having an SRH of very good/excellent at any time point seems to be associated with a decreased risk of VTE among women. The clinical implication of the present study could be to include recurring assessments of SRH in primary care.

### Supplementary Information

Below is the link to the electronic supplementary material.

Supplementary file1 (DOCX 34 KB)

## Data Availability

Due to ethical and legal restrictions related to the Swedish Biobanks in Medical Care Act (2002:297) and the Personal Data Act (1998:204), data are available upon request from the data access group of MPP Study by contacting Anders Dahlin (anders.dahlin@med.lu.se).
