# Supplementary material for: Association between changed self-rated health and the risk of venous thromboembolism in Malmö Preventive Program: a cohort study
Source: J Thromb Thrombolysis. 2024 Jan 24;57(3):497–502. doi: 10.1007/s11239-023-02933-4 (PMC10961270; doi:10.1007/s11239-023-02933-4)
Supplement: Supplementary file 1 — Supplementary file1 (DOCX 34 KB) [file 11239_2023_2933_MOESM1_ESM.docx]

Supplementary

**Table 1**. The present study includes the following ICD-codes

| ICD-7 | 463-466,583.00, 334.40,334.50, 682, 684 |
| --- | --- |
| ICD-8 | 321, 450-453, 671 (not 671.00), 673.9 |
| ICD-9 | 437G, 451-453, 415B, 416B, 416W, 671C, D, E, F, X,673C, 639G |
| ICD-10 | I26, I636, I676, I80-82, O222-225, O229, O870-873, O879, O882, O082, O087 |

**Table 2**. Group comparison between characteristics of individuals with incident or no incident VTE.

|  | **Men** n (%) | | **Women** n (%) | |
| --- | --- | --- | --- | --- |
|  | No incident VTE | Incident VTE | No incident VTE | Incident VTE |
| **SRH**** (re-examination)  Very Poor Poor Good Very good Excellent Missing | 76 (1.0) 750 (10.2) 3376 (46.0) 2063 (28.1) 1080 (14.7) 24 | 9 (1.6) 53 (9.2) 283 (49.1) 158 (27.4) 74 (12.8) 2 | 75 (1.6) 680 (14.7) 2458 (53.1) 1033 (22.3) 387 (8.4) 30 | 5 (1.2) 71 (17.6) 219 (54.2) 82 (20.3) 27 (6.7) 1 |
| **SRH***** (re-examination)  Very good/excellent Poor/fair Missing | 3142 (42.7) 4202 (57.0) 24 | 232 (40.1) 345 (59.6) 2 | 1420 (30.5) 3213 (68.9) 31 | 109 (26.9) 295 (72.8) 1 |
| **SRH****** (baseline)  Good Poor Missing | 5761 (78.2) 1608 (21.8) 0 | 469 (81.0) 110 (19) 0 | 3379 (73.6) 1213 (26.4) 72 | 268 (67.3) 130 (32.7) 7 |
| **SRH change**  Poor to Poor/fair Poor to Very good/excellent Good to Poor/fair Good to very good/excellent Missing | 1782 (24.2) 1361 (18.5) 3000 (40.7) 1226 (16.6) 0 | 141 (24.4) 91 (15.7) 261 (45.1) 86 (14.9) 0 | 879 (19.1) 535 (11.5) 2159 (47.0) 1022 (22.2) 69 | 120 (30.2) 35 (8.8) 172 (43.2) 71 (17.8) 7 |
| **Age** (re-examination)  No. Mean Std deviation | 7369 67.2 6.0 | 579 68.2 5.8 | 4664 69.0 4.9 | 405 70.4 4.4 |
| **Height (cm)** (re-examination)  No. Mean Std deviation | 7348 175.66 6.67 | 573 176.70 6.57 | 4646 162.12 5.95 | 402 162.50 6.30 |
| **Weight (kg)** (re-examination)  No. Mean  Std deviation | 7345 83.71 12.67 | 574 85.99 13.47 | 4641 70.23 12.19 | 400 74.41 13.39 |
| **BMI (m^2^/kg)** (re-examination)  No. Mean Std deviation | 7342 27.11 3.69 | 573 27.53 3.91 | 4641 26.73 4.52 | 399 28.21 5.11 |
| **Hip circumference** (re-examination)  No. Mean Std deviation | 7336 102.1 7.14 | 571 103.1 7.04 | 4636 103.1 9.20 | 404 105.9 11.04 |
| **Waist circumference** (re-examination)  No. Mean Std deviation | 7339 98.4 10.17 | 573 100.2 10.71 | 4638 86.6 11.64 | 404 90.5 12.78 |
| **Systolic blood pressure** (re-examination)  No. Mean Std deviation | 7298 146.4 19.98 | 570 146.2 19.31 | 4616 144.2 20.85 | 404 146.0 22.73 |
| **Diastolic blood pressure** (re-examination) No. Mean Std deviation | 7298 84.7 10.80 | 570 84.6 10.36 | 4615 82.14 10.21 | 404 82.9 11.48 |
| **Alcohol consumption^** re-examination  No. Mean Std deviation | 7091 118.6 137.2 | 556 132.4 178.2 | 4179 56.7 89.5 | 357 48.1 71.6 |
| **Smoking** (re-examination)  Yes Sometimes Former smoker No Missing | 1084 (15.1) 276 (3.8) 3552 (49.4) 2283 (31.7) 174 | 74 (13.3) 19 (3.4) 304 (54.5) 160 (28.7) 22 | 605 (13.3) 132 (2.9) 1459 (32.0) 2368 (51.9) 100 | 42 (10.6) 8 (2.01) 142 (35.7) 206 (51.8) 7 |
| **Hormone substitution^^** (re-examination)  No Yes Missing | 7366 (99.97) 2 (0.03) 1 | 579 (100.0) 0 0 | 4052 (86.9) 612 (13.1) | 353 (87.2) 52 (12.8) |
| **Prevalent varicose veins** (re-examination)  No Yes Missing | 7347 (99.7) 22 (0.3) 0 | 577 (99.7) 2 (0.3) 0 | 4620 (99.1) 44 (0.9) 0 | 396 (97.8) 9 (2.2) 0 |

*Without missing, **SRH on a five-step scale, at re-examination. *** SRH after dichotomisation at re-examination with Good SRH= Very good or good, Poor SRH= good, poor or very poor. **** Do you feel perfectly healthy; Yes (Good SRH) No (Poor SRH) baseline. ^Self-reported average alcohol consumption grams per week at re-examination, ^^ hormonal replacement therapy at re-examination

**Table 3**. Univariate Cox-regression with hazard ratios for VTE in relation to assumed risk factors.

|  | **Hazard Ratio** | **95% CI** | **n** | **Failures** |
| --- | --- | --- | --- | --- |
| **SRH change** (baseline and re-examination) ***Men*** *Good to very good/excellent Good to Poor/fair Poor to Very good/excellent Poor to Poor/fair* | 1.01 1.16 .98 Ref. | .71 ; 1.42 .85 ; 1.58 .68 ; 1.40 | 7937 | 354 |
| **SRH change** (baseline and re-examination) ***Women*** *Good to very good/excellent Good to Poor/fair Poor to very good/excellent Poor to Poor/fair* | .52 .65 .46 Ref | .36 ; .76 .49 ; .86 .28 ; .74 | 4990 | 265 |
| **Very good/excellent SRH** (re-examination)  Men Women | .89 .69 | .72 ; 1.10 .52 ; .91 | 7912 5034 | 354 268 |
| **Good SRH** (baseline)  Men Women | 1.16 .66 | .89 ; 1.51 .52 ; .85 | 7937 4987 | 3554 265 |
| **Age** (re-examination)  Men Women | 1.04 1.06 | 1.02 ; 1.05 1.03 ; 1.09 | 7937 5066 | 354 269 |
| **BMI** (re-examination)  Men Women | 1.03 1.07 | 1.01 ; 1.06 1.05 ; 1.10 | 7905 5037 | 351 266 |
| **Height (cm)** (re-examination)  Men Women | 1.03 1.00 | 1.01 ; 1.04 .98 ; 1.03 | 7911 5045 | 351 267 |
| **Weight (kg)** (re-examination)  Men Women | 1.02 1.03 | 1.01 ; 1.02 1.02 ; 1.04 | 7909 5038 | 351 267 |
| **Hip circumference** (re-examination)  Men Women | 1.03 1.03 | 1.01 ; 1.04 1.02 ; 1.04 | 7897 5038 | 351 269 |
| **Waist circumference** (re-examination)  Men Women | 1.02 1.03 | 1.01 ; 1.03 1.02 ; 1.04 | 7902 5040 | 352 269 |
| **High SBP (mmHg)***(re-examination)  Men Women | 0.88 1.31 | .71 ; 1.08 1.03 ; 1.67 | 7859 5017 | 350 269 |
| **High DBP (mmHg)****(re-examination)  Men Women | .97 1.28 | 0.76 ; 1.24 .96 ; 1.70 | 7859 5017 | 350 269 |
| **Smoking** (ref non-smoker) (re-examination)  Men *Current Sometimes Former* | 1.07 .64 1.17 | .75 ; 1.50 .31 ; 1.31 .92 ; 1.49 | 7742 | 343 |
| **Smoking** (ref non-smoker) (re-examination)  Women *Current Sometimes Former* | .89 .69 1.07 | .60 ; 1.34 .28 ; 1.67 .82 ; 1.39 | 4959 | 263 |
| **Alcohol consumption** (re-examination)  ***Men***  *0 g/week 0-12 g/week 12-130 g/week >130 g/week* | 1.04 .23 Ref 1.07 | .77 ; 1.43 .03 ; 1.67  .85 ; 1.35 | 7637 | 339 |
| **Alcohol consumption** (re-examination)  ***Women*** *0 g/week 0-12 g/week 12-130 g/week >130 g/week* | 1.04 1.22 Ref .98 | .79 ; 1.37 .62 ; 2.41  .65 ; 1.50 | 4533 | 238 |
| ***Men*** *Sedentary leisure time Moderate leisure time physical activity Regular physical activity and training Hard training or competitive sports* | .87 .91 Ref .84 | .55 ; 1.34 .70 ; 1.18  .11 ; 6.04 | 7868 | 351 |
| **Exercise** (re-examination)  ***Women*** *Sedentary leisure time Moderate leisure time physical activity Regular physical activity and training Hard training or competitive sports* | 1.20 .95 Ref 1.52e-18 | .76 ; 1.91 .69 ; 1.30  . | 5017 | 263 |
| **Prevalent varicose veins** (re-examination)  Men Women | 1.63 2.18 | .81 ; 3.29 1.28 ; 3.74 | 7937 5066 | 354 269 |

**Table 4**. Sensitivity analysis, univariate Cox-regression with hazard ratios for assumed risk factors

|  | **Hazard Ratio** | **95% CI** | **p-value** | **n** | **Failures** |
| --- | --- | --- | --- | --- | --- |
| **SRH change**  *Men* *Good to Very good/excellent Good to Poor/fair Poor to Very good/excellent Poor to Poor/fair*  *Women* *Good to Very good/excellent Good to Poor/fair Poor to Very good/excellent Poor to Poor/fair* | .98 1.11 .98 Ref.  .55 .69 .50 Ref | .71 ; 1.35 .83 ; 1.48 .70 ; 1.37    .38 ; .79 .52 ; .91 .32 ; .80 | 0.910 0.475 0.904    **0.001 0.008 0.003** | 9295  5368 | 398  282 |
| **Very good/excellent SRH (re-examination)**  Men Women | .91 .70 | .74 ; 1.11 .54 ; .92 | .332 **.011** | 9264 5411 | 398 286 |
| **Good SRH (baseline)**  Men Women | 1.21 .77 | .99 : 1.46 .63 ; .95 | .051 .012 | 9313 5369 | 680 432 |
| **Age**  Men Women | 1.03 1.06 | 1.02 ; 1.05 1.03 ; 1.08 | **.000 .000** | 9295 5451 | 398 287 |
| **BMI**  Men Women | 1.01 1.07 | .99 ; 1.02 1.05 ; 1.10 | **.201 .000** | 9250 5415 | 395 284 |
| **Height (cm)**  Men Women | 1.03 1.01 | 1.01 ; 1-04 .99 ; 1.03 | **.001** .476 | 9260 5425 | 395 285 |
| **Weight (kg)**  Men Women | 1.01 1.03 | 1.00 ; 1.02 1.02 ; 1.04 | **.002 .000** | 9256 5418 | 395 285 |
| **Hip**  Men Women | 1.02 1.03 | 1.01 ; 1.03 1.02 ; 1.04 | **.006 .000** | 9247 5417 | 394 287 |
| **Waist**  Men Women | 1.01 1.03 | 1.00 ; 1.02 1.02 ; 1.04 | **.003 .000** | 9254 5420 | 395 287 |
| **High SBP (mmHg)***  Men Women | 0.86 1.33 | .70 ; 1.05 1.05 ; 1.69 | .132 .017 | 9201 5395 | 393 287 |
| **High DBP (mmHg)****  Men Women | .97 1.28 | 0.77 ; 1.22 .97 ; 1.68 | .769 .076 | 9201 5395 | 393 287 |
| **Smoking** (ref non-smoker)  Men *Current Sometimes Former*  Women *Current Sometimes Former* | 1.02 .60 1.16  .92 .63 1.10 | .73 ; 1.41 .30 ; 1.18 .92 ; 1.45  .63 ; 1.36 .26 ; 1.53 .85 ; 1.42 | .916 .139 .208  .686 .304 .467 | 9053  5331 | 385  280 |
| **Alcohol consumption**  Men  *0 g/week 0-12 g/week 12-130 g/week >130 g/week*  Women *0 g/week 0-12 g/week 12-130 g/week >130 g/week* | 1.11 .41 Ref 1.07  1.00 1.15 Ref .98 | .83 ; 1.47 .10 ; 1.66  .86 ; 1.34  .79 ; 1.37 .62 ; 2.41  .65 ; 1.46 | .488 .213  .544  .999 .694  907 | 8936  4872 | 382    253 |
| **Exercise** (ref none) Men *Sedentary leisure time Moderate leisure time physical activity Regular physical activity and training Hard training or competitive sports*  Women *Sedentary leisure time Moderate leisure time physical activity Regular physical activity and training Hard training or competitive sports* | .99 .88 Ref 1.51  1.33 .99 Ref 1.59e-18 | .68 ; 1.45 .69 ; 1.12  .37 ; 6.12  .55 ; 2.07 .72 ; 1.35  . | .969 .302  .567  .194 .944  . | 9208  5393 | 395  281 |
| **Prev. varicose veins**  Men Women | 1.79 2.27 | .95 ; 3.35 1.37 ; 3.76 | .070 **.001** | 9295 5451 | 398 287 |
| Exclusions of patients at baseline with deep venous thrombosis, pulmonary embolism, portal vein thrombosis, superficial thrombophlebitis, malignancy or treated with warfarin medication. *Ref <140 mmHg, ** Ref < 90 mmHg. In sensitivity analysis there were 685 failures of a total 14769 observations, 23 ended on or before entering. The total time at risk were 145,423.7 person-time during a follow-up time of 16.81 years. Corresponding to an incident rate of 4.71 (95% CI 4.37 – 5.07) | | | | | |

**Table 5**. Sensitivity analysis, multivariate Cox regression with hazard ratios and adjustments for confounding variables

| **Model** | **1** | **2** | **3** |
| --- | --- | --- | --- |
| **SRH change**  *Men* Good to Very good/excellent Good to Poor/fair Poor to Very good/excellent Poor to Poor/fair  Total no  Failures no | 1.01 (95% CI .73 ; 1.39) p= .935 1.09 (95% CI .82 ; 1.46) p= .392 1.02 (95% CI .72 ; 1.42) p= .940  Ref 9295 398 | 1.04 (95% CI .75 ; 1.44) p= .804 1.11 (95% CI .83 ; 1.49) p= .480 1.02 (95% CI .72 ; 1.44) p= .980  Ref  9052 385 | 1.06 (95% CI .81 ; 1.41) p= .649 1.20 (95% CI .95 ; 1.53) p= .120 1.15 (95% CI .86 ; 1.54) p= .335  Ref  9310 565 |
| **SRH change**  *Women* Good to Very good/excellent Good to Poor/fair Poor to Very good/excellent Poor to Poor/fair  Total no  Failures no | .58 (95% CI .40 ; .84) p= .004 .69 (95% CI .53 ; .91) p= .008 .54 (95% CI .34 ; .85) p= .008 Ref 5368 282 | .57 (95% CI .40 ; .83) p= .003  .70 (95% CI .53 ; .92) p= .010 .50 (95% CI .31 ; .81) p= .005  Ref  5252 275 | .73 (95% CI .51 ; .97) p= .034  .72 (95% CI .56 ; .89) p= .004 .55 (95% CI .33 ; .80) p= .003  Ref  5385 374 |
| Adjustments | Age | Age, smoking | Age, smoking, waist circumference, varicose veins |
| Proportional hazard assumption, global test Men | .3124 | .1328 | .3318 |
| Proportional hazard assumption, global test Women | .9980 | .8070 | .8885 |

Exclusions of patients at baseline with deep venous thrombosis, pulmonary embolism, portal vein thrombosis, superficial thrombophlebitis, malignancy or treated with warfarin medication.
